# Non-beverage alcohol consumption among individuals experiencing chronic homelessness in Edmonton, Canada: a cross-sectional study

**DOI:** 10.1186/s12954-021-00555-8

**Published:** 2021-10-17

**Authors:** Jean Nicolas Westenberg, Mostafa Mamdouh Kamel, Sindi Addorisio, Mohammad Abusamak, James S. H. Wong, Ava Outadi, Kerry L. Jang, R. Michael Krausz

**Affiliations:** 1 Department of Psychiatry, University of British Columbia (UBC), David Strangway Building, 5950 University Boulevard, Vancouver, BC V6T 1Z3 Canada; 2 Department of Psychiatry, Tanta University, Tanta, Egypt; 3 School of Public Health, University of Alberta, Edmonton, AB Canada; 4 Faculty of Dentistry, McGill University, Montreal, QC Canada

**Keywords:** Non-beverage alcohol, Homelessness, Harm reduction, Perceived needs, High-risk drinking, Managed alcohol program

## Abstract

**Background:**

Among individuals experiencing homelessness, the prevalence of alcohol use disorder is extremely high. Alcohol-related harms are compounded by the use of non-beverage alcohol (NBA; e.g. rubbing alcohol, cooking wine). The dangers of NBA consumption pose significant risks to the individual and to others when consumed in large quantities and when mixed with other substances. The objectives of this paper are to describe the alcohol consumption patterns of individuals experiencing homelessness, identify substance use patterns, psychological stressors, and related harms associated with NBA consumption, and compare NBA consumers to non-NBA consumers in relation to their use of services and perceived barriers to care.

**Methods:**

Using a cross-sectional survey, 150 individuals experiencing homelessness were recruited from Edmonton’s inner city and adjoining areas. Frequency, quantity, and volume of alcohol consumption were used to assess patterns of alcohol use in the last 6 months. Descriptive statistics and bivariate analyses were used to compare participants reporting NBA consumption and non-NBA consumption (*p* ≤ 0.05).

**Results:**

The majority of participants were male (71.3%) and self-identified as Indigenous (74.0%). Overall, 24% (*n* = 36) reported NBA consumption within the last six months. NBA consumers were older than non-NBA consumers (*p* = 0.005), reported different perceived living stability (*p* = 0.022), and had higher psychological distress (*p* = 0.038). The majority of NBA consumers reported not receiving harm reduction services while also not needing such services (*n* = 18, 51.4%), which differed from non-NBA consumers (*p* = 0.003). Structural barriers (e.g. availability, location, cost) were most frequently reported as reasons for unmet harm reduction (60.9%) and hospital care (58.3%) needs, while barriers to skills training (58.5%) and counselling services (53.6%) were mostly motivational (e.g. personal beliefs).

**Conclusions:**

Within such an already marginalized population experiencing homelessness, individuals who consume NBA represent a vulnerable subpopulation who require adapted and distinct health and social services to stabilize and recover. Current harm reduction services are not prepared to effectively assist this group of individuals, and specific treatment programs are rare. Managed alcohol programs are a feasible approach but must be tailored to the specific needs of those who consume NBA, which is especially important for Indigenous people. More comprehensive assessments of NBA consumption are needed for program development and policy recommendations.

**Supplementary Information:**

The online version contains supplementary material available at 10.1186/s12954-021-00555-8.

## Background

Individuals experiencing homelessness and who are dependent on alcohol are known to experience higher levels of chronic illness, concurrent substance use, mental health disorders, injuries, assaults, longer hospital stays, and higher levels of contact with the criminal justice system than the general population [1, 2]. Moreover, people experiencing unstable housing have a much higher mortality rate from alcohol-related causes than that of the general population [3]. An 11-year follow-up study found mortality ratios of 6.4 for men and 8.5 for women for alcohol-related deaths when comparing individuals living in shelters, rooming houses, and hotels with the national cohort [3]. Socially marginalized drinkers also face structural barriers to achieving good overall health care in the community, leading to a disproportionately high level of emergency service utilization, most often in the form of transportation to emergency departments by first responders [4–6]. For instance, one study found that individuals experiencing chronic homelessness and alcohol dependence presented at the emergency department unconscious roughly four times more than general homeless population and individuals in low-income housing [5].

Problems related to alcohol dependence in populations experiencing homelessness are compounded by the use of non-beverage alcohol (NBA) such as rubbing alcohol, mouthwash, cooking wine, and cologne. NBAs are broadly understood to be liquids with extremely high alcohol content that are not intended for human consumption [7–10]. One standard alcoholic drink in Canada contains 17.05 mL (or 13.45 g) of pure ethanol, which translates into 12 oz of beer (5% alcohol by volume; ABV), 5 oz of wine (12% ABV), 1.5 oz of distilled spirits (40% ABV) [11]. In comparison, one Canadian standard alcoholic drink of hand sanitizer (62% ABV) is less than 1 oz of solution, and the consumption of one 500 ml bottle of 95% rubbing alcohol is the equivalent of about 28 standard drinks [12, 13]. The high concentration of alcohol found in NBA poses serious risks of injury, disease and death, and can result in significant detrimental health effects [14, 15]. Additive ingredients and denaturing agents in NBA such as methanol or isopropanol can also be particularly hazardous to health when consumed in large doses, but the ethanol remains the most toxic and high-risk component of NBA [7, 16].

The most common reasons individuals turn to NBA are affordability, effects and availability. Since NBAs are manufactured alcohol-based products and not classed as alcoholic beverages, they avoid excise duty which are put in place to discourage the consumption of goods that are deemed to be harmful to consumers' health [17, 18]. The combined lower cost and high percentage alcohol content create an incentive for people who are dependent on alcohol and have difficulty affording beverage alcohol [19]. For example, 99% isopropyl alcohol is sold for less than 3 times the price of the cheapest vodkas (40% ABV) in Canada [20, 21]. Moreover, NBA is readily available and can be accessed in settings where alcoholic beverages are otherwise unavailable such as in hospitals, prisons, nursing homes, or in military settings [7, 10, 18].

In Edmonton, Alberta, Canada, the number of individuals experiencing homelessness is on the rise, as is high-risk drinking in the community. Only a limited number of shelters welcome individuals who consume NBA due to stigma, often requiring abstinence during stay, and only a small proportion of NBA consumers are provided with much-needed access to different types of supports such as food, clothing, counselling or health care [22, 23]. Individuals who consume NBA represent a subpopulation amidst homeless communities who experience both severe alcohol dependence and housing instability.

Although much research has focused on alcohol and drug use among vulnerable individuals experiencing homelessness, minimal research has specifically focused on NBA consumption. Despite it being recorded in all regions of the world, most research on unrecorded alcohol (any alcohol produced and/or consumed that is not officially registered, including NBA) comes from Russia and former parts of the Soviet Union [24–26]. For instance, a survey among adults in Izhevsk, Russia, reported a high prevalence of NBA consumption (28%), and sociodemographic characteristics of NBA consumers reported in the study included being single, unemployed or retired, younger than 19 or older than 29 years, having lower educational status, and having lower income [27]. In North America, previous studies have mostly comprised of semi-structured qualitative interviews with members of managed alcohol programs (MAPs) and community leaders, who have provided insight into the alcohol-related harms experienced, and have reported the impacts of various care services on alcohol consumption patterns, physical and mental health, quality of life, etc. [9, 12, 28–31]. These studies have been essential in optimizing service delivery and developing programs tailored to their needs. However, most participants in these studies have been recruited by virtue of their program involvement and their established contact with harm reduction services. Moreover, most of the available literature on individuals experiencing homelessness have failed to distinguish NBA consumers from non-NBA consumers, thereby seldom recognizing the unique health and social service needs of NBA consumers in particular. Therefore, little is known about individuals who consume NBA, including their perceived needs and barriers to care, and especially among individuals experiencing homelessness not already in touch with MAPs.

The objectives of this paper are to describe the alcohol consumption patterns (beverage and non-beverage) of individuals experiencing street homelessness in Edmonton, Canada, and to identify the substance use patterns, psychological stressors, and related harms associated with NBA consumption. This is an important first step in obtaining a quantitative picture of individuals who consume NBA and experiencing chronic homelessness in Canada. In addition, this paper aims to compare NBA consumers to non-NBA consumers in relation to their use of and access to services, as well as their perceived barriers to care. To our knowledge, this is the first Canadian study to concentrate on NBA consumption using a quantitative approach and to thoroughly describe a subpopulation of individuals who consume NBA, within a larger community of individuals experiencing long-term homelessness in Canada.

## Methods

### Study design

The Undiagnosed Mental Illness in Individuals Experiencing Homelessness (UMIIEH) study was a cross-sectional survey conducted between November 2017 and June 2018 in Edmonton, Alberta, Canada [32]. The UMIIEH study utilized a structured survey instrument, including 57 single, multi-item, and brief qualitative measures divided into five sections: (1) demographics, (2) living situation, (3) substance use, (4) physical and mental health, (5) health service utilization, needs, and barriers. The full survey can be found in the appendix (Additional File 1). The study protocol received ethical approval from the University of Alberta’ Health Research Ethics Board. This study is reported following the Strengthening the Reporting of Observational Studies in Epidemiology (STROBE) guidelines (Additional File 3) [33].

As this study involves a community composed primarily of individuals who identify as Indigenous, the research team consulted the Elder of the community for cultural guidance and proper protocol, and was invited to a pipe ceremony in which the Elder blessed the study and participants [34]. Moreover, this study recognizes that reporting on data from Indigenous people is not neutral and can be reported in ways that reinforces settler colonialism of the continued oppression of Indigenous peoples [34]. However, the Elder advising on this project believe this information is important to the community and useful for resource allocation.

### Recruitment

Participants were recruited by convenience sampling from Edmonton’s inner city, adjoining parkland areas, and river valley. Interviews were carried out by trauma-informed, culturally sensitive, trained research staff who had formed strong and welcoming relationships with the Elder and the community. To be eligible for the study, participants had to report experiencing absolute homelessness in Edmonton within the last 6 months, defined as camping or “sleeping rough” in parkland/river valley areas or staying on the street with infrequent shelter stay. Individuals were recruited by the Street Outreach workers from Boyle Street Community Services and the 24/7 Crisis Diversion Team (CDT). The Outreach staff started recruitment at 7am, usually ending at 3 pm, hiking in the river valley and parklands. Outreach staff already had established relationships with individuals within the community and were therefore well received and invited into tents or shelters to conduct the survey. The 24/7 CDT would recruit from streets, doorways and bus shelters, starting at 3 pm and ending at 3am. The majority of recruitment was done during the winter months with temperatures averaging minus 20–30 °C (minus 4–22°F). Once participants provided their written consent, surveys were completed in about 15–30 min. Participants were provided with a $20 cash honorarium upon survey completion. Similarly, Indigenous cultural protocols were followed and small pouches of tobacco were offered to participants, as recommended and guided by the community Elder advising on this project. Data were collected from 150 individuals; one individual’s data were excluded partway through due to delayed onset of intoxication.

### Measures of alcohol consumption

To identify current alcohol drinkers, participants were asked whether they had had a drink containing alcohol in the last 6 months. If their response was affirmative, they were then asked what they consumed (beers, wine, hard liquor) and how much of each, which was later converted to standard drinks. One drink was defined as 12 oz of beer, 5 oz of wine, and 1.5 oz of hard liquor.

Participants were considered NBA consumers if they responded "yes" to the following question “In the last 6 months, did you drink cooking wine, rubbing alcohol/mouthwash, or cologne?" A similar process was done for quantity of NBA consumed. All questions regarding alcohol consumption are on pages 3–4 of the survey (Additional File 1).

### Measures of service utilization and barriers to care

Health service utilization was assessed using an adapted version of the Perceived Need for Care Questionnaire (PNCQ) [35]. Participants were asked if they had received hospital care, counselling, skilling training or harm reduction services in the past 12 months. Participants could respond: “no, I did not need this kind of help [no need]”, “yes, I received this kind of help in the past 12 months [received]”, “yes, I received this kind of help in the past 12 months but not as much service as I needed [underserved]”, or “no, but I think I needed this kind of help in last 12 months [unserved]”.

### Measures of barriers to care

Participants who reported having perceived unmet service needs in the past 12 months were asked to specify one or more reasons for their unmet needs. Participants were given the option to choose from a close-ended list of reasons or provide an open-ended ‘other’ verbatim response. Answers were then classified as motivational barriers or structural barriers. Motivational barriers included the following “I prefer to manage my own care”, “I didn't think anything would help”, “I was afraid to ask for help or what others might think of me”, and “I did not want to get help at the time”, while structural barriers included the following “I didn't know where to find help”, “I couldn’t financially afford the treatment”, “I asked for help, but did not receive it”, “the wait-list was too long/there was no space available for me”, “I was only allowed a limited amount of time”.

### Statistical analyses

Descriptive statistics were used for demographic information, substance use patterns, mental and physical health status, as well as service use and needs. Bivariate analyses including the Kruskal–Wallis rank sum test and the Pearson's Chi-squared test were used to compare participants reporting NBA consumption and non-NBA consumption in relation to the study covariates, such as mean age, sex, and ethnicity, and the independent variables, such as self-reported housing situation, substance use, psychological symptom severity, psychological distress, health-related quality of life, as well as service use and needs. These analyses provide a comprehensive description of NBA consumption and of individuals who consume NBA, in order to help us understand who is most likely to use NBA and how to better address the needs and preferences of those who do. However, these analyses do not imply causation by controlling for potentially confounding variables in a multivariate analysis, which would not be appropriate given the small sample size and the numerous variables. The statistical program R 3.61 was used for all analyses and a significance level of 0.05 [36].

## Results

### Alcohol consumption

Overall, almost all participants (*n* = 150, 88%) reported consuming alcohol within the last 6 months (Table 1). Of those who consumed alcohol, more than half reported having an alcoholic drink four or more times a week (*n* = 70, 46.7%), while only a quarter reported drinking alcohol once a month or less (*n* = 34, 22.7%). When participants were asked how many alcoholic drinks they had on a typical day of drinking, nearly half reported 10 or more drinks (*n* = 64, 42.7%). When participants were asked how frequently they drink five or more alcoholic drinks on one occasion, half reported daily or almost daily (*n* = 67, 44.7%).

**Table 1:** Descriptive statistics of alcohol consumption among individuals experiencing absolute homelessness

Characteristics	Total sample
Beverage alcohol consumption (*n* = 150); *n* (%)	
Yes	132 (88)
No	18 (12)
Frequency of beverage alcohol consumption (*n* = 132); *n* (%)	
Once a month or less	34 (22.7)
Two to four times a month	15 (10)
Two to three times a week	12 (8)
Four or more times a week	70 (46.7)
Number of drinks on a typical day (*n* = 131); *n* (%)	
1 or 2	25 (16.7)
3 or 4	12 (8)
5 or 6	17 (11.3)
7 or 9	13 (8.7)
10 or more	64 (42.7)
Frequency of 5 drinks or more (*n* = 131); *n* (%)	
Never	16 (10.7)
Less than monthly	20 (13.3)
Monthly	17 (11.3)
Weekly	11 (7.3)
Daily or almost daily	67 (44.7)
NBA consumption (*n* = 149); *n* (%)	
Yes	36 (24)
No	113 (76)
Frequency of NBA consumption (*n* = 36); *n* (%)	
Once a month or less	13 (36.1)
2–4 times a month	7 (19.4)
2–3 times a week	3 (8.3)
4 or more times a week	13 (36.1)
Number of NBA drinks on a typical day (*n* = 27); mean (IQR)	2.5 (3)
Most NBA drinks on a typical day (*n* = 27); mean (IQR)	4 (11)
Physical violence after drinking (*n* = 111); *n* (%)	
Yes, in last 6 months	41 (36.9)
Yes, but not in last 6 months	18 (16.2)
No	52 (46.8)

About a quarter of the sample reported NBA consumption within the last six months (*n* = 36, 24%) (Table 1). Among those that consumed NBA, the frequency ranged from once a month or less (*n* = 13, 36.1%) to 4 or more times per week (*n* = 13, 36.1%). The number of NBA drinks consumed during a typical day of drinking ranged from 1 to 32 (median = 2.5, IQR = 3), and the most consumed in one day ranged from 1 to 64 drinks (median = 4, IQR = 10).

### Demographics of NBA consumers

The mean age of NBA consumers was 46.28 (SD = 7.77). The large majority of them were male (*n* = 30, 83.3%) and self-identified as Indigenous, First Nations, Metis, or Inuit (*n* = 32, 88.9%). NBA consumers mainly lived on the street or in parks (*n* = 15, 41.7%; *n* = 14, 38.9%), and the majority lived alone (*n* = 31, 86.1%). The majority of NBA consumers had been in their living situation for over 18 months (*n* = 20, 55.6%). When asked about their current living situation, three quarters reported having a very unstable housing situation (*n* = 27, 75%) and reported being very dissatisfied with it (*n* = 24, 66.7%).

The differences in demographic characteristics between NBA consumers and non-NBA consumers are presented in Additional File 2: Table S1. NBA consumers were significantly older than non-NBA consumers (*p* = 0.005) and reported significantly different perceived living stability (*p* = 0.022). Proportionally more NBA consumers lived alone, were male, and self-identified as Indigenous relative to non-NBA consumers; however, these did not reach statistical significance.

### Substance use among NBA consumers

Of the 36 NBA consumers, 71.4% reported illicit substance use in the past 6 months (*n* = 26). Stimulant use seemed to be the most prevalent among NBA consumers: 69.4% reported stimulant use (*n* = 25), while only 22.5% reported opioid use in the last 6 months (*n* = 8). Only 7 NBA consumers reported using both stimulants and opioids (19.4%). Injection drug use was quite low in this subpopulation (*n* = 8, 22.9%). Methamphetamine was the most commonly used stimulant (*n* = 23, 63.9%), followed by crack cocaine (*n* = 8, 22.2%), and cocaine (*n* = 7, 19.4%). The type of opioid used was diverse with the exception of fentanyl, which was reportedly not used by any NBA consumer. The patterns of substance use were not significantly different between NBA and non-NBA consumers (Additional File 2: Table S2).

### Physical and mental health of NBA consumers

When assessing the psychological symptom severity of NBA consumers, the majority reported being lonely (*n* = 22, 62.9%), nervous/worried/frustrated (*n* = 20, 57.1%), and depressed (*n* = 19, 54.3%) at least every day. Almost half reported having racing thoughts (*n* = 17, 48.6%), feeling suspicious or paranoid (*n* = 16, 45.7%), and having trouble concentrating/remembering (*n* = 15, 42.9%) at least every day (Additional File 2: Table S3). Using the Kessler Psychological Distress Scale to measure of psychological distress in the past 30 days, 77.1% scored greater than or equal to 13, indicating a high probability of nonspecific psychological distress. The majority reported feeling worthless (*n* = 14, 40.0%), nervous (*n* = 12, 34.3%), and hopeless (*n* = 10, 28.6%) all the time. NBA consumers had significantly higher levels of psychological distress when compared to non-NBA consumers (*p* = 0.038), as measured by the mean Kessler Psychological Distress score (Additional File 2: Table S3). Health-related quality of life as measured by the EQ-5D-3L demonstrated that almost all of the NBA consumers reported problems with their health, but this seemed widespread to the entire sample as no statistical significance was reached (Additional File 2: Table S3).

### Service use among NBA and non-NBA consumers

**Fig. 1:**
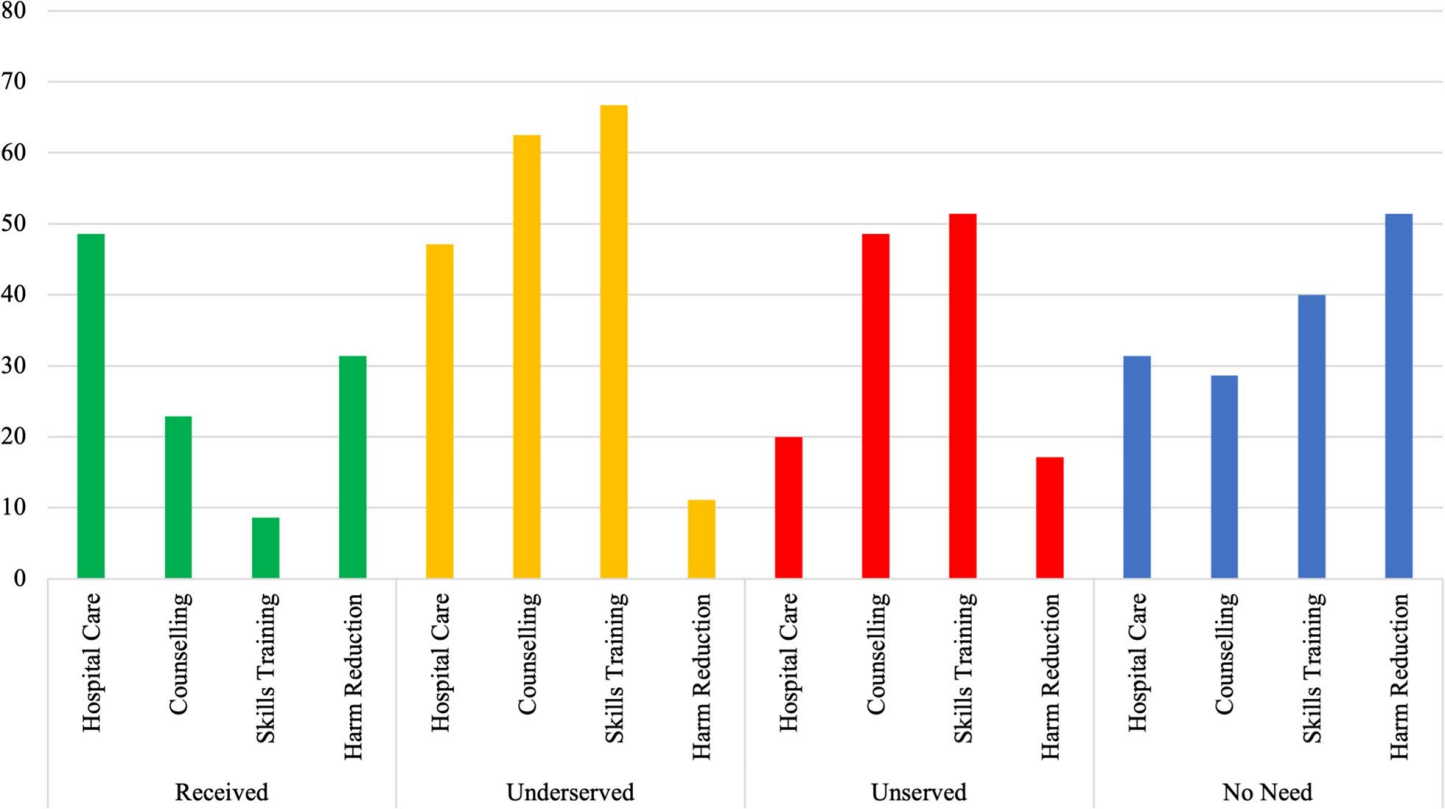
Received, underserved, unserved, and unneeded services among NBA consumers experiencing homelessness.* Legend*: Health service utilization was assessed using an adapted version of the Perceived Need for Care Questionnaire (PNCQ). Participants could respond: “no, I did not need this kind of help [no need]”, “yes, I received this kind of help in the past 12 months [received]”, “yes, I received this kind of help in the past 12 months but not as much service as I needed [underserved]”, or “no, but I think I needed this kind of help in last 12 months [unserved]”

A summary of the use of and need for general health and social services during the past year regarding substance use and/or mental health problems is summarized in Additional File 2: Table S4. Almost half of NBA consumers reported utilizing hospital services (*n* = 17, 48.6%), which is proportionally more than non-NBA consumers but not significantly different. Of those that received hospital care, roughly 47.1% felt like their needs had not been met (underserved need, *n* = 8) (Fig. 1). Skills training and counselling were the most unserved needs: 51.4% and 48.6% thought they needed skills training and counselling services, respectively, but had not sought these services (*n* = 18; *n* = 17). The least needed service as perceived by NBA consumers was harm reduction: 51.4% reported not receiving harm reduction services and not needing this kind of help (*n* = 18). The use and need for harm reduction services were significantly different between the two subpopulations (*p* = 0.003) (Additional File 2: Table S4).

### Perceived barriers to care among NBA and non-NBA consumers

**Fig. 2:**
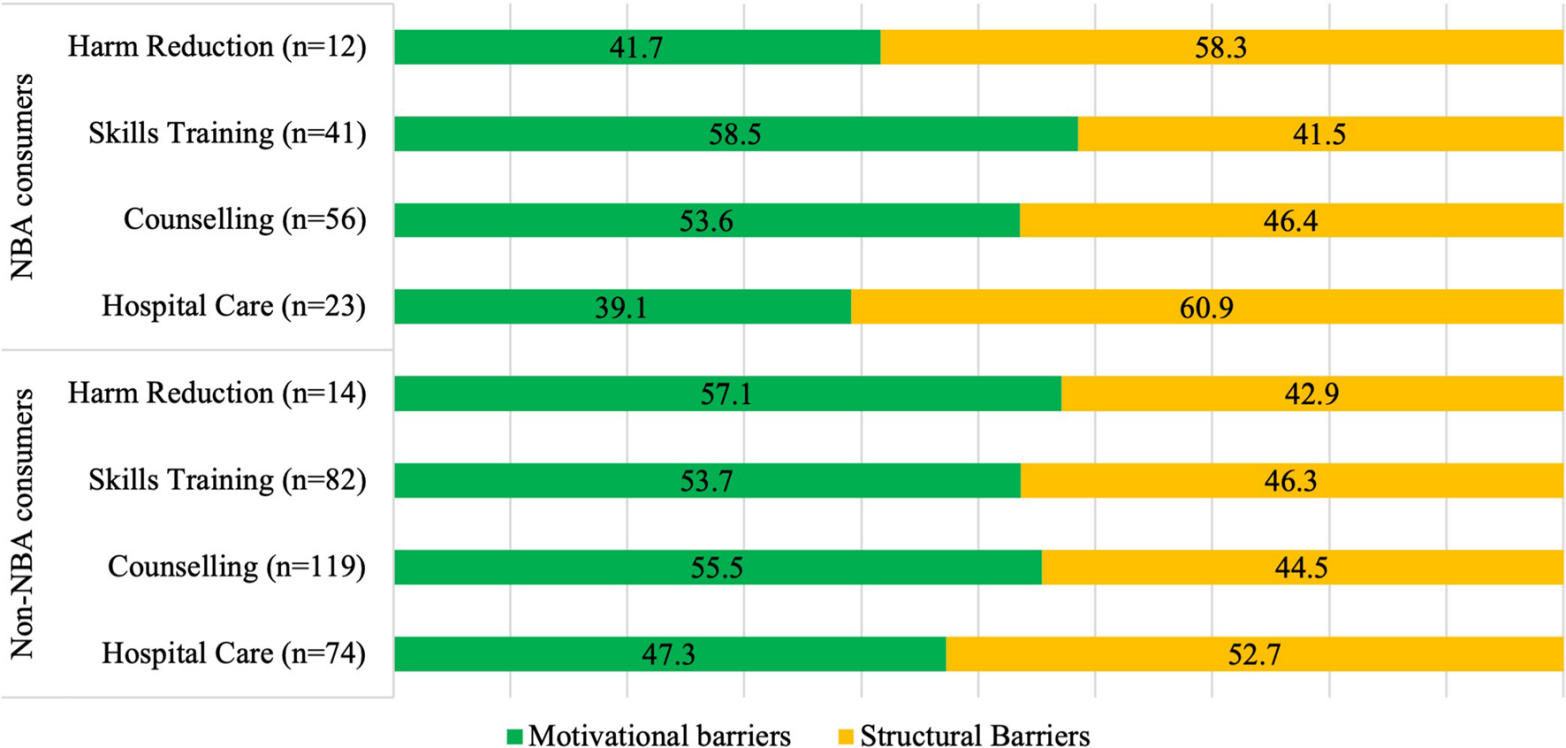
Self-reported reasons for perceived unmet need for care across health and social services. * Legend*: Motivational barriers included the following “I prefer to manage my own care”, “I didn't think anything would help”, “I was afraid to ask for help or what others might think of me”, and “I did not want to get help at the time.” Structural barriers included the following “I didn't know where to find help”, “I couldn’t financially afford the treatment”, “I asked for help, but did not receive it”, “the wait-list was too long/there was no space available for me”, “I was only allowed a limited amount of time”

For NBA consumers, structural barriers were the most frequently endorsed reasons for unmet harm reduction and hospital care needs, while barriers to skills training and counselling services were motivational for the majority (Fig. 2). For non-NBA consumers, hospital care was the only service in which structural barriers were most frequently endorsed reasons for unmet needs. All other service needs, including counselling, skills training, and harm reduction, were unmet due to motivational barriers (Fig. 2).

## Discussion

In this study, we examined beverage and non-beverage alcohol consumption among 150 individuals experiencing street homelessness in Edmonton, Canada. The sample represents a community of individuals that have been sleeping unsheltered despite being in one of the coldest cities in Canada, where temperatures often fall below − 20 °C (− 4°F) during the winter. Similar to other homeless populations, this cohort is characterized by poor physical and mental health as well as prevalent alcohol and substance misuse [37]. Almost all participants reported consuming alcohol within the last 6 months, and nearly half of all participants reported heavy drinking or binge drinking daily or almost daily. NBA consumption was also quite prevalent, with 1 in every 4 individuals reporting NBA consumption within the last 6 months. The quasi-absence of available resources for a highly vulnerable population living in an extreme environment leads to the most high-risk drinking pattern observed, with the intent of quick intoxication thereby reaching threatening blood alcohol levels. These patterns of consumption are as dangerous as injection drug use and come with physically damaging withdrawal syndromes with a high likelihood of adverse events. It is surprising that these individuals get so little attention. Although there are a limited number of studies examining NBA consumption, similar proportions have been reported in street-entrenched individuals in Russia and Australia [27, 38]. Within this sample, NBA consumers were older, experienced greater social and physical instability, and reported increased levels of psychological distress including symptoms of suspicion, paranoia, nervousness, worry, and frustration. They all reported unique service needs, specifically for harm reduction services to help cope with strong withdrawal symptoms and the physical consequences of their use. The level of needs among individuals who consume NBA is no different from marginalized people who inject drugs.

Of particular interest is the finding that NBA consumers perceive structural barriers to meeting their harm reduction service needs, whereas non-NBA consumers perceive the barriers to be motivational. These findings might underlie a sentiment that harm reduction services for those that primarily consume alcohol are lacking, as they are often more tailored to illicit drug users (safe injection sites, needle exchange, drug testing, etc.). Valid evidence on harm reduction services for alcohol use is limited, especially in relation to supervised consumption facilities [39].

Similar to other harm reduction initiatives, managed alcohol programs (MAPs) allow onsite alcohol consumption and inebriation, help manage alcohol use with provision of regulated daily doses of beverage alcohol, and provide access to other basic determinants of health such as housing, health care and social supports [28, 30]. Preliminary research from Canada has shown that MAP participation has been associated with a number of positive outcomes including fewer hospital admissions, detox episodes, police contacts leading to custody, reduced NBA consumption, and reductions of alcohol-related harms including legal issues, home life, and withdrawal seizures [12, 28–30, 40, 41]. Currently there are approximately 23 MAPs across Canada, the majority of which are residential, providing some type of accommodation in a shelter, transitional, or permanent supportive housing [42]. Though there exist differences between residential and community MAPs, the overarching goals of all involve preserving dignity, reducing harms of drinking, and increasing access to housing, health, and social services [43]. The perceived harms of alcohol use by socially marginalized drinkers have much in common with those of other illicit substances, but some substance-specific harms including dangers of withdrawal, unintentional injuries, violence and theft are unique to illicit drinkers [9]. MAPs can help people experiencing long-term homelessness and severe alcohol dependence reduce alcohol-related harms, promote service engagement, counter marginalization, and develop an environment favourable for their recovery and reintegration [9]. Emergency housing and/or shelters without integrated access to MAPs, or that restrict entrance to those who are using or not sober, are characterized by displacement and survival in high-risk environments for people experiencing chronic homelessness and severe alcohol dependence [30, 44]. The abstinence-based, pre-MAP arena is also marked by multiple losses and intense exclusion and disconnection from family and friends [30, 44]. Given their effectiveness, harm reduction services such as MAPs must be developed and implemented according to the specific needs of those experiencing long-term homelessness and consuming NBA. This is especially important for Indigenous individuals. MAPs have largely been incompatible with Indigenous cultural practices and traditions, and must therefore expand beyond risk reduction and develop into places of healing, safety, recovery and reconnection.

Hospital services were substantially more utilized than any other service, demonstrating how disconnected and segregated this population is in the system of care. Individuals who consume NBA are disproportionately barred from shelters, clinics, housing projects, community centres, grocery stores and even public spaces, becoming arguably the most street entrenched demographic in the inner city. The high prevalence of aggression and violence associated with homelessness reported in this study and in the literature further the stigma and widen the gap in care and from the community [45–48]. Though heavy drinking and intoxication have also been associated with violence and aggression, people who self-identify as consumers of NBA have linked much of this violence to exploitation and lack of access to housing, since individuals who become overly intoxicated or pass out alone in public are taken advantage of and are at risk of theft and sexual assault [9, 49, 50]. Supervised alcohol consumption spaces have been suggested as possible ways of reducing related harms, similar to supervised injection sites for people who inject drugs [9].

Finally, the impacts of settler colonialism, assimilative health policies and anti-Indigenous racism have resulted in significant health inequities for Indigenous Peoples [51]. Indigenous people are stigmatized and pathologized when coping behaviours, such as the consumption of alcohol, are examined from a decontextualized perspective. Substance use and mental health issues resulting from ongoing colonization are prevalent in First Nations, Inuit and Métis peoples. Our sample, composed of participants primarily identifying as Indigenous, reported very high rates of frequent and heavy alcohol use. Similarly, in a community-based study including data from 735 Indigenous people from 8 reserves, diagnostic assessments revealed a lifetime alcohol abuse prevalence of 50.4% and lifetime alcohol dependence of 24.4% [52]. In the literature, strong motives to drink have been associated with stronger First Nations identity and greater perceived Indigenous specific racism [53]. Moreover, poverty is an Indigenous determinant of health due to access to resources on occupied land, and this influences the types of alcohol consumed by First Nations, especially NBA [54, 55]. Almost all NBA consumers in our sample self-identified as Indigenous, First Nations, Metis, or Inuit. Indigenous culture and intergenerational cohesion must be prioritized in targeted prevention, early intervention, and treatment approaches that are non-stigmatic, accessible, and supportive of Indigenous wholistic health practices [51, 54–57].

It is evident that non-beverage alcohol and its variations (surrogate alcohol, illicit alcohol and unrecorded alcohol) require further attention from the research and clinical community [58]. Historically, consumption of NBA has been predominantly discussed in the context of alcohol policy, which has resulted in mainly political or legislative responses. Foul-tasting or toxic denaturing products are added to make these products unfit for drinking and to deter human consumption [59]. Methanol, for instance, is a traditional denaturing agent which appears in NBAs such as anti-freeze, paint solvents, and windshield washer fluid, but can pose serious health threats including blindness and death if consumed in large quantities [7, 17, 60]. More comprehensive assessments of NBA consumption and the associated health and social service needs are critical to program development and policy recommendations in order to optimize service delivery, improve quality of life, and help support stability and housing [26, 32, 61].

### Limitation

This study has limitations. The findings are based on a cross-sectional survey done in one city, and therefore, caution is needed when extrapolating these findings across different homeless populations or across different time points as they may not be generalizable [62]. Moreover, although several factors may have contributed to our results. Our sample, albeit unique, is relatively small and renders multivariate analysis challenging, thereby not taking into account risk factors such as onset and extent of alcohol use, prevalence of comorbid mental disorders, and particular social factors (e.g. poor housing, poverty). Thus, any causal interpretations are cautioned, and further investigation in subsequent more highly powered studies is needed. A longitudinal cohort would be a significant next step in examining temporal relationships and better exploring the associations between NBA consumption and other variables.

### Conclusions

Individuals who consume NBA represent a marginalized group that is ignored in the system of care. Within populations experiencing homelessness, individuals who consume NBA are stigmatized and the target of significant mistreatment, violence, and criminalization. They are underrecognized in program development and in the clinical literature, despite the high prevalence of mental health, physical health, and substance use challenges within this subpopulation, and the dire need for adapted health and social services. Current harm reduction services are not well prepared to deal with NBA consumption effectively, and specific treatment programs are a rare exception. MAPs are an appropriate approach for this vulnerable subpopulation but must be tailored to their needs. Individualizing programs and services to create spaces of cultural safety, recovery, healing, and reconnection are especially important for Indigenous people. The high prevalence of NBA consumption among Indigenous individuals emphasizes the necessity of supporting Indigenous communities and developing embedded supports in remote areas.

## Supplementary Information

**Additional file 1**. Undiagnosed Mental Illness in Individuals Experiencing Homelessness (UMIIEH) Survey

**Additional file 2**.** Table S1**: Differences among individuals who consume NBA and those who do not.** Table S2**: Substance use among NBA consumers relative to non-NBA consumers.** Table S3**: Physical and mental health among NBA consumers relative to non-NBA consumers.** Table S4**: Use and need for care for one or more general health and social services during the past year regarding substance use and/or mental health problems

**Additional file 3**. STROBE Checklist

## Data Availability

The datasets generated during and/or analysed during the current study are available from the corresponding author on reasonable request.

## Abbreviations

NBA: Non-beverage alcohol
ABV: Alcohol by volume
MAP: Managed alcohol programs
UMIIEH: Undiagnosed Mental Illness in Individuals Experiencing Homelessness
STROBE: Strengthening the Reporting of Observational Studies in Epidemiology
CDT: Crisis Diversion Team
PNCQ: Perceived Needs for Care Questionnaire
