# Supplementary material for: Non-beverage alcohol consumption among individuals experiencing chronic homelessness in Edmonton, Canada: a cross-sectional study
Source: Harm Reduct J. 2021 Oct 17;18:108. doi: 10.1186/s12954-021-00555-8 (PMC8522138; doi:10.1186/s12954-021-00555-8)
Supplement: Supplementary file 1 — Additional file 1. Undiagnosed Mental Illness in Individuals Experiencing Homelessness (UMIIEH) Survey [file 12954_2021_555_MOESM1_ESM.docx]

| Study participant ID: |  |
| --- | --- |
| Interview date (DD/MM/YYYY): | / / |
| Participant recruited from: |  |
| Interviewer initials: |  |
| Interview start time: | AM / PM |

| INTERVIEW SCRIPT  Thank you for contributing to this study. As we go through the interview together, please keep in mind that there are no wrong answers. It’s very important that you answer as honestly as you can. We rely on your information to help create positive change for people who are experiencing homelessness.  We realize some of these questions are sensitive. If you do not want to answer a question, just let me know and we will move on. It is better for you to refuse to answer a question than to give a false answer. False answers affect the quality of our data and limit our ability to advocate for positive change.  We take your privacy very seriously. All the information that you provide will only be kept between you, me and the research team. We never report any individual information.  If there are any questions you don’t understand, please stop me and ask for clarification. The interview takes about 35 mins. If you need a break, let me know and we can stop for a short rest before we finish the interview. |
| --- |

| **Demographics** |
| --- |

**1. How old are you?** _________________ years old

**2.** **What is your gender identity?**

- Male
- Female
- Transgender
- Other

**3. What ethnic group do you identify with?** __________________________

| **Now I will be asking you about your living situation.** |
| --- |

1. **Where are you currently staying?**

- Parkland ( River Valley, Millcreek, Rundle Park etc.)
- Shelter
- Street
- Couchsurfing
- Other

1. **How long have you been staying there?**

- 0-6 months
- 6-12 months
- 12-18months
- More than 18 months

1. **Who are you living with?**
2. **How would you describe your living situation?**

- Very unstable
- A little unstable
- Neutral – neither unstable nor stable
- A little stable
- Very stable

1. **How satisfied are you with your current living situation?**

- Very unsatisfied
- A little unsatisfied
- Neutral – neither satisfied nor unsatisfied
- A little satisfied
- Very satisfied

| *Now, I would like to ask you about your consumption of drinks containing alcohol and drug use.* |
| --- |
|  |

**1**. **Have you had a drink containing alcohol in the last 6 months? One drink meaning 12oz beer, 5oz wine, 1.5oz hard liquor.**

- Yes
- No **(Go to Q 6)**

*If they answer yes, proceed with these questions.  If not, skip to the next main section.*

**2.** **How often do you have a drink containing alcohol?**

- Once a month or less
- Two to four times a month
- Two to three times a week
- Four or more times a week
- Don't know
- Refused

**3.**  **How many drinks containing alcohol do you have on a typical day when you are drinking?**

- 1 or 2
- 3 or 4
- 5 or 6
- 7 to 9
- 10 or more
- Don’t know
- Refused

**4.** **How often do you have five or more drinks on one occasion?**

- Never
- Less than monthly
- Monthly
- Weekly
- Daily or almost daily
- Don’t know​
- Refused

**5. In the last 6 months, did you drink cooking wine, rubbing alcohol/ mouthwash, or cologne?**

- - Yes
  - No **(Go to Q.6)**
  - Don't know
  - Refused

**a. How often would you drink these?**

- - 4 or more times per week
  - 2-3 times a week
  - 2-4 times a month
  - Once a month or less
  - Don't Know
  - Refused

**b. In the last 6 months, how much cooking wine, rubbing alcohol/ mouthwash, or cologne did you drink during one typical day?**

- - **
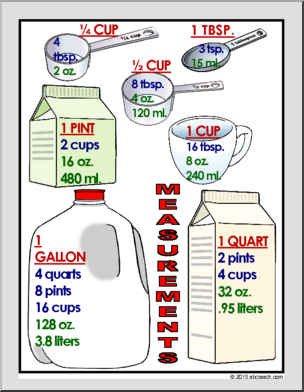
**_________ (# of drinks)
  - Don’t know
  - *Refused*

1. **In the last 6 months, what was *the most* that you drank in one day cooking wine, rubbing alcohol/mouthwash, or cologne?**
   - _________ (# of drinks)
   - Don’t know
   - *Refused*

**d. In the last week how many days did you drink cooking wine, rubbing alcohol/mouthwash, or cologne**?

- - __________(# of ***days***)
  - Don't know
  - Refused

**e. In the past 6 months have you ever become physically violent while you were under the influence of alcohol?**

- Yes
- No
- Yes, but not in the last 6 months
- Yes, longer than 6 months ago
- Don't know
- Refused

| *The next few questions are about any illicit stimulant drug use* |
| --- |

**6. In the last 6 months have you used any illegal stimulants? (ie; crystal meth)**

- Yes
- No **(Go to Q.8)**

**7. In the last 6 months what have you used? (check all that apply)**

|  | Crack Cocaine |
| --- | --- |
|  | Cocaine (sniffed or snorted) |
|  | Crystal Meth (smoked) (pint, speed) |
|  | Crystal Meth (snorted) |
|  | Crack Cocaine (inject) |
|  | Cocaine (inject) |
|  | Crystal meth (inject) |
|  | Other (name) |

**8. In the last 6 months have you used any illegal opioids? (ie; heroin, fentanyl)**

- **Yes**
- **No (Go to Q 10)**

| Heroin (sniffed or snorted) |
| --- |
| Heroin (smoked) |
| Heroin (inject) |
| Dilaudid (inject) |
| OxyCotin (inject) |
| Morphine (inject) |
| Fentanyl (inject) |
| Other (specify) |

**8. Do you have a Naloxone Kit?**

- Yes
- No **(Go to Q.10)**
- Don't Know
- Refused

**9**. **Have you used it in the past 6 months?**

- Yes
- No
- Don't Know
- Refused

a) If yes, how many times? _________________

| *Now, I would like to ask you about your state of health.* |
| --- |

**10. Place a tick in one box in each category: Which statement best describes your own health state?**

Mobility

- I have no problems walking
- I have some problems walking
- I am limited to a wheelchair or walker

Self-Care (hygiene, washing and clothing oneself...)

- I have no problems with self-care
- I have some problems washing and/or dressing myself
- I am unable to wash or dress myself without assistance

Usual Activities (leisure, work, cleaning...)

- I have no problems with performing my daily activities
- I have some problems with performing my daily activities
- I am unable to perform my activities without assistance

Pain/Discomfort

- I have no pain or discomfort
- I have moderate pain or discomfort
- I have extreme pain or discomfort

Anxiety/Depression

- I am not anxious or depressed
- I am moderately anxious or depressed
- I am extremely anxious or depressed

| *Now, I would like to ask you about your mental health and level of formal healthcare.* |
| --- |

**11. In the past month, how often have you felt nervous, worried, or frustrated?**

- Not at all
- Once during the month
- Several times during the month
- Several times a week
- At least every day
- Don't know
- Declined

**12. In the past month, how often have you felt depressed?**

- Not at all
- Once during the month
- Several times during the month
- Several times a week
- At least every day
- Don't know
- Declined

**13. In the past month, how often have you felt lonely?**

- Not at all
- Once during the month
- Several times during the month
- Several times a week
- At least every day
- Don't know
- Declined

**14. In the past month, how often did you hear voices, or hear or see things that other people didn't think were there?**

- Not at all
- Once during the month
- Several times during the month
- Several times a week
- At least every day
- Don't know
- Declined

**15. In the past month, how often did you have trouble making up your mind, or deciding about something?**

- Not at all
- Once during the month
- Several times during the month
- Several times a week
- At least every day
- Don't know
- Declined

**16. In the past month, how often did you have trouble thinking straight, concentrating, or remembering?**

- Not at all
- Once during the month
- Several times during the month
- Several times a week
- At least every day
- Don't know
- Declined

**17. In the past month, how often did you have problems with thinking too fast (thoughts racing)?**

- Not at all
- Once during the month
- Several times during the month
- Several times a week
- At least every day
- Don't know
- Declined

**18. In the past month, how often did you feel suspicious or paranoid?**

- Not at all
- Once during the month
- Several times during the month
- Several times a week
- At least every day
- Don't know
- Declined

**19.** **In the past 12 months, have you been formally diagnosed with a mental health problem or long-term psychological issue by a health care professional?**

- Yes, I have been diagnosed by a professional in the last 12 months
- Yes, I have been diagnosed, but *not* within the last 12 months
- No, I have never been diagnosed by a professional
  1. If you were diagnosed in the last 12 months, were you prescribed any medication(s) for psychological issues?
- Yes, I was prescribed medications, and I used them
- Yes, I was prescribed medication, but I did *not* use them
- No, I was not prescribed medications

**20. In the past 12 months, have you received hospital care (overnight or longer) because of problems with your emotions, mental health, or use of drugs and/or alcohol? (eg. treating an infection, overdose, psychosis…)**

- Yes, I have received care in the past 12 months
- No, but I think I needed this kind of help, but did not seek it
- No, I did not need this kind of help
- *Refused to answer*
  1. Do you think you got as much hospital care as you needed?
- Yes
- No
  1. Please indicate if any of the following reasons stopped you from receiving any or enough hospital care in the past 12 months:
- I prefer to manage my own care
- I didn’t think anything would help
- I didn’t know where to find help
- I was afraid to ask for help, or what others might think of me
- I couldn’t financially afford the treatment
- I asked for help, but did not receive it
- I did not want to get help at the time
- The wait-list was too long/there was no space available for me
- I was only allowed a limited amount of time in the hospital

**21. In the past 12 months, have you received counselling (outside of a hospital including any kind of help to talk through your problems) because of problems with your emotions, mental health, or use of drugs and/or alcohol?**

- Yes, I have received care in the past 12 months
- No, but I think I needed this kind of help, but did not seek it
- No, I did not need this kind of help
- *Refused to answer*
  1. Do you think you got as much counselling as you needed?
- Yes
- No
  1. Please indicate if any of the following reasons stopped you from receiving any or enough counselling in the past 12 months:
- I prefer to manage my own care
- I didn’t think anything would help
- I didn’t know where to find help
- I was afraid to ask for help, or what others might think of me
- I couldn’t financially afford the treatment
- I asked for help, but did not receive it
- I did not want to get help at the time
- The wait-list was too long/there was no space available for me
- I was only allowed a limited amount of time for counselling

**22. In the past 12 months, have you received skills training (help to improve your ability to work, to care for yourself, to meet people) because of problems with your emotions, mental health, or use of drugs and/or alcohol?**

- Yes, I have received care in the past 12 months
- No, but I think I needed this kind of help, but did not seek it
- No, I did not need this kind of help
- *Refused to answer*
  1. Do you think you got as much skills training as you needed?
- Yes
- No
  1. Please indicate if any of the following reasons stopped you from receiving any or enough skills training in the past 12 months:
- I prefer to manage my own care
- I didn’t think anything would help
- I didn’t know where to find help
- I was afraid to ask for help, or what others might think of me
- I couldn’t financially afford the treatment
- I asked for help, but did not receive it
- I did not want to get help at the time
- The wait-list was too long/there was no space available for me
- I was only allowed a limited amount of time for skills training

**23. In the past 12 months, have you received access to harm reduction services (clean needle exchange) to reduce the risk of harm related to using drugs because of problems with your emotions, mental health, or use of drugs and/or alcohol?**

- Yes, I have received care in the past 12 months
- No, but I think I needed this kind of help, but did not seek it
- No, I did not need this kind of help
- *Refused to answer*
  1. Do you think you got as much harm reduction services as you needed?
- Yes
- No
  1. Please indicate if any of the following reasons stopped you from receiving any or enough harm reduction services in the past 12 months:
- I prefer to manage my own care
- I didn’t think anything would help
- I didn’t know where to find help
- I was afraid to ask for help, or what others might think of me
- I couldn’t financially afford the treatment
- I asked for help, but did not receive it
- I did not want to get help at the time
- The wait-list was too long/there was no space available for me
- I was only allowed a limited amount of time in the hospital

**Kessler 6 Scale: Quantifies non-specific psychological distress**

**In the past 4 weeks (28 days), how much of the time did you feel…**

|  | All of the time | Most of the time | Some of the time | A little of the time | None of the time |
| --- | --- | --- | --- | --- | --- |
| So sad nothing could cheer you up? | 1 | 2 | 3 | 4 | 5 |
| Nervous? | 1 | 2 | 3 | 4 | 5 |
| Restless or fidgety? | 1 | 2 | 3 | 4 | 5 |
| Hopeless? | 1 | 2 | 3 | 4 | 5 |
| That everything was an effort? | 1 | 2 | 3 | 4 | 5 |
| Worthless? | 1 | 2 | 3 | 4 | 5 |
|  |  |  |  |  |  |

**24. Do you have a confidant or someone you trust that you talk to?**

- Yes
- No
- Don't know

**25. What do you think about the development of Rogers Place?**

**26. Has the development of the arena impacted your access to services?**

- Yes
- No
- Don't know

If yes, can you tell me in what ways?

**27. Do you see any added daily barriers due to the arena?**

- Yes
- No
- Don't Know

If Yes, give example.

**28. Do you avoid coming into the downtown area now that the area is built?**

- Yes
- No
- Don't KNow

**We would like to thank you for your participation in this study, your story is valued.**

**Research Team**
