# Supplementary material for: Non-beverage alcohol consumption among individuals experiencing chronic homelessness in Edmonton, Canada: a cross-sectional study
Source: Harm Reduct J. 2021 Oct 17;18:108. doi: 10.1186/s12954-021-00555-8 (PMC8522138; doi:10.1186/s12954-021-00555-8)
Supplement: Supplementary file 2 — Additional file 2. Table S1: Differences among individuals who consume NBA and those who do not. Table S2: Substance use among NBA consumers relative to non-NBA consumers. Table S3: Physical and mental health among NBA consumers relative to non-NBA consumers. Table S4: Use and need for care for one or more general health and social services during the past year regarding substance use and/or mental health problems [file 12954_2021_555_MOESM2_ESM.docx]

Table 1. Demographic differences between individuals who consume NBA and those who do not, within a sample of individuals experiencing absolute homelessness

| Characteristics | | NBA Consumers (n=36) | Non-NBA consumers (n=113) | Significance | Total Sample (n=150) |
| --- | --- | --- | --- | --- | --- |
| Age; mean (SD)* | | 46.28 (7.77) | 41.10 (10.05) | **0.005** | 42.30 (9.76) |
| Gender; n (%) | |  |  |  |  |
|  | Male | 30 (83.3) | 77 (68.1) | 0.121 | 107 (71.3) |
|  | Female | 6 (16.7) | 36 (31.9) |  | 43 (28.7) |
| Ethnicity; n (%) | |  |  |  |  |
|  | Caucasian | 4 (11.1) | 32 (28.6) | 0.084 | 37 (24.8) |
|  | Indigenous | 32 (88.9) | 79 (70.5) |  | 111 (74.5) |
|  | Middle Eastern | 0 (0.0) | 1 (0.9) |  | 1 (0.7) |
| Place of stay; n (%) | |  |  |  |  |
|  | Parkland | 14 (38.9) | 49 (43.4) | 0.689 | 64 (42.7) |
|  | Shelter | 7 (19.4) | 22 (19.5) |  | 29 (19.3) |
|  | Street | 15 (41.7) | 39 (34.5) |  | 54 (36.0) |
|  | Other | 0 (0.0) | 3 (2.7) |  | 3 (2.0) |
| Length of stay; n (%) | |  |  |  |  |
|  | 0-6 months | 5 (13.9) | 15 (13.3) | 0.617 | 20 (13.3) |
|  | 6-12 months | 8 (22.2) | 15 (13.3) |  | 23 (15.3) |
|  | 12-18 months | 3 (8.3) | 10 (8.8) |  | 13 (8.7) |
|  | More than 18 months | 20 (55.6) | 73 (64.6) |  | 94 (62.7) |
| Living arrangement; n (%) | |  |  |  |  |
|  | Alone | 31 (86.1) | 72 (63.7) | 0.059 | 103 (68.7) |
|  | Partner | 3 (8.3) | 24 (21.2) |  | 28 (18.7) |
|  | Immediate family | 1 (2.8) | 2 (1.8) |  | 3 (2.0) |
|  | Friend | 1 (2.8) | 15 (13.3) |  | 16 (10.7) |
| Stability of living situation; n (%)* | |  |  |  |  |
|  | Very unstable | 27 (75.0) | 63 (55.8) | **0.022** | 91 (60.7) |
|  | A little unstable | 0 (0.0) | 15 (13.3) |  | 15 (10.0) |
|  | Neutral | 4 (11.1) | 22 (19.5) |  | 26 (17.3) |
|  | A little stable | 0 (0.0) | 6 (5.3) |  | 6 (4.0) |
|  | Very stable | 5 (13.9) | 7 (6.2) |  | 12 (8.0) |
| Satisfaction with living situation; n (%) | |  |  |  |  |
|  | Very unsatisfied | 24 (66.7) | 70 (61.9) | 0.111 | 95 (63.3) |
|  | A little unsatisfied | 1 (2.8) | 12 (10.6) |  | 13 (8.7) |
|  | Neutral | 4 (11.1) | 18 (15.9) |  | 22 (14.7) |
|  | A little satisfied | 2 (5.6) | 9 (8.0) |  | 11 (7.3) |
|  | Very satisfied | 5 (13.9) | 4 (3.5) |  | 9 (6.0) |

Table 2. Differences in substance use patterns between individuals who consume NBA and those who do not, within a sample of individuals experiencing absolute homelessness

| Characteristics | | NBA Consumers (n=36) | Non-NBA consumers (n=113) | Significance |
| --- | --- | --- | --- | --- |
| IV behavior; n (%) | |  |  |  |
|  | Yes | 8 (22.9) | 46 (40.7) | 0.769 |
|  | No | 37 (77.1) | 67 (59.3) |  |
| Illicit Substance use; n (%) | |  |  |  |
|  | Neither | 10 (27.8) | 24 (21.2) | 0.566 |
|  | Both | 7 (19.4) | 36 (31.9) |  |
| Overall stimulants use; n (%) | |  |  |  |
|  | Yes | 25 (69.4) | 86 (76.1) | 0.563 |
|  | No | 11 (30.6) | 27 (23.9) |  |
| Crack cocaine use; n (%) | |  |  |  |
|  | Yes | 8 (22.2) | 25 (22.1) | 1 |
|  | No | 28 (77.8) | 88 (77.9) |  |
| Cocaine use; n (%) | |  |  |  |
|  | Yes | 7 (19.4) | 25 (22.1) | 0.914 |
|  | No | 29 (80.6) | 88 (77.9) |  |
| Meth use; n (%) | |  |  |  |
|  | Yes | 23 (63.9) | 75 (66.4) | 0.943 |
|  | No | 13 (36.1) | 38 (33.6) |  |
| Stimulant Injection; n (%) | |  |  |  |
|  | Yes | 9 (25.0) | 37 (32.7) | 0.504 |
|  | No | 27 (75.0) | 76 (67.3) |  |
| Overall opioid use; n (%) | |  |  |  |
|  | Yes | 8 (22.9) | 39 (34.5) |  |
|  | No | 27 (77.1) | 74 (65.5) | 0.277 |
| Heroin use; n (%) | |  |  |  |
|  | Yes | 4 (11.4) | 23 (20.4) | 0.371 |
|  | No | 31 (88.6) | 90 (79.6) |  |
| Dilaudid use; n (%) | |  |  |  |
|  | Yes | 4 (11.4) | 19 (16.8) | 0.616 |
|  | No | 31 (88.6) | 94 (83.2) |  |
| Oxycontin use; n (%) | |  |  |  |
|  | Yes | 3 (8.6) | 9 (8.0) | 1 |
|  | No | 32 (91.4) | 104 (92.0) |  |
| Morphine use; n (%) | |  |  |  |
|  | Yes | 2 (5.7) | 14 (12.4) | 0.424 |
|  | No | 33 (94.3) | 99 (87.6) |  |
| Fentanyl use; n (%) | |  |  |  |
|  | Yes | 0 (0.0) | 11 (9.7) | 0.121 |
|  | No | 35 (100.0) | 102 (90.3) |  |
| Other opioids use; n (%) | |  |  |  |
|  | Yes | 2 (5.7) | 9 (8.0) | 0.94 |
|  | No | 33 (94.3) | 104 (92.0) |  |
| Opioid Injection; n (%) | |  |  |  |
|  | Yes | 4 (11.4) | 29 (25.7) | 0.7 |
|  | No | 31 (88.6) | 84 (74.3) |  |

Table 3. Differences in physical and mental health between individuals who consume NBA and those who do not, within a sample of individuals experiencing absolute homelessness

| Characteristics | | NBA Consumers (n=36) | Non-NBA consumers (n=113) | Significance |
| --- | --- | --- | --- | --- |
| Nervous/worried/frustrated; n (%)* | |  |  |  |
|  | Not at all | 6 (17.1) | 8 (7.3) | **0.033** |
|  | Once during the month | 0 (0.0) | 10 (9.1) |  |
|  | Several times during the month | 5 (14.3) | 22 (20.0) |  |
|  | Several times a week | 4 (11.4) | 27 (24.5) |  |
|  | At least every day | 20 (57.1) | 43 (39.1) |  |
| Depression; n (%) | |  |  |  |
|  | Not at all | 7 (20.0) | 21 (18.8) | 0.067 |
|  | Once during the month | 4 (11.4) | 10 (8.9) |  |
|  | Several times during the month | 4 (11.4) | 26 (23.2) |  |
|  | Several times a week | 1 (2.9) | 18 (16.1) |  |
|  | At least every day | 19 (54.3) | 37 (33.0) |  |
| Lonely; n (%) | |  |  |  |
|  | Not at all | 5 (14.3) | 18 (16.2) | 0.5 |
|  | Once during the month | 2 (5.7) | 10 (9.0) |  |
|  | Several times during the month | 5 (14.3) | 17 (15.3) |  |
|  | Several times a week | 1 (2.9) | 12 (10.8) |  |
|  | At least every day | 22 (62.9) | 54 (48.6) |  |
| Auditory/Visual Hallucinations; n (%) | |  |  |  |
|  | Not at all | 20 (57.1) | 67 (61.5) | 0.286 |
|  | Once during the month | 2 (5.7) | 6 (5.5) |  |
|  | Several times during the month | 3 (8.6) | 20 (18.3) |  |
|  | Several times a week | 5 (14.3) | 6 (5.5) |  |
|  | At least every day | 5 (14.3) | 10 (9.2) |  |
| Indecisiveness; n (%) | |  |  |  |
|  | Not at all | 14 (40.0) | 37 (32.7) | 0.585 |
|  | Once during the month | 0 (0.0) | 4 (3.5) |  |
|  | Several times during the month | 6 (17.1) | 24 (21.2) |  |
|  | Several times a week | 3 (8.6) | 16 (14.2) |  |
|  | At least every day | 12 (34.3) | 32 (28.3) |  |
| Thinking Straight; n (%) | |  |  |  |
|  | Not at all | 10 (28.6) | 34 (30.6) | 0.766 |
|  | Once during the month | 3 (8.6) | 7 (6.3) |  |
|  | Several times during the month | 4 (11.4) | 19 (17.1) |  |
|  | Several times a week | 3 (8.6) | 14 (12.6) |  |
|  | At least every day | 15 (42.9) | 37 (33.3) |  |
| Racing Thoughts; n (%) | |  |  |  |
|  | Not at all | 9 (25.7) | 41 (36.6) | 0.21 |
|  | Once during the month | 2 (5.7) | 8 (7.1) |  |
|  | Several times during the month | 6 (17.1) | 9 (8.0) |  |
|  | Several times a week | 1 (2.9) | 12 (10.7) |  |
|  | At least every day | 17 (48.6) | 42 (37.5) |  |
| Suspicious/Paranoid; n (%)* | |  |  |  |
|  | Not at all | 10 (28.6) | 51 (45.9) | **0.014** |
|  | Once during the month | 2 (5.7) | 4 (3.6) |  |
|  | Several times during the month | 6 (17.1) | 17 (15.3) |  |
|  | Several times a week | 1 (2.9) | 17 (15.3) |  |
|  | At least every day | 16 (45.7) | 22 (19.8) |  |
| Feeling so sad that nothing could cheer them up; n (%)* | |  |  |  |
|  | All of the time | 4 (11.4) | 25 (22.1) | **0.002** |
|  | Most of the time | 3 (8.6) | 25 (22.1) |  |
|  | Some of the time | 8 (22.9) | 37 (32.7) |  |
|  | A little of the time | 7 (20.0) | 13 (11.5) |  |
|  | None of the time | 13 (37.1) | 13 (11.5) |  |
| Feeling nervous; n (%)* | |  |  |  |
|  | All of the time | 12 (34.3) | 32 (28.3) | **0.027** |
|  | Most of the time | 2 (5.7) | 31 (27.4) |  |
|  | Some of the time | 5 (14.3) | 23 (20.4) |  |
|  | A little of the time | 7 (20.0) | 13 (11.5) |  |
|  | None of the time | 9 (25.7) | 14 (12.4) |  |
| Feeling restless or fidget; n (%)* | |  |  |  |
|  | All of the time | 7 (20.0) | 25 (22.1) | **0.012** |
|  | Most of the time | 2 (5.7) | 22 (19.5) |  |
|  | Some of the time | 3 (8.6) | 27 (23.9) |  |
|  | A little of the time | 8 (22.9) | 11 (9.7) |  |
|  | None of the time | 15 (42.9) | 28 (24.8) |  |
| Feeling hopeless; n (%) | |  |  |  |
|  | All of the time | 10 (28.6) | 35 (31.0) | 0.073 |
|  | Most of the time | 5 (14.3) | 14 (12.4) |  |
|  | Some of the time | 3 (8.6) | 30 (26.5) |  |
|  | A little of the time | 8 (22.9) | 10 (8.8) |  |
|  | None of the time | 9 (25.7) | 24 (21.2) |  |
| Feeling everything was an effort; n (%) | |  |  |  |
|  | All of the time | 7 (20.0) | 20 (17.7) | 0.735 |
|  | Most of the time | 2 (5.7) | 16 (14.2) |  |
|  | Some of the time | 10 (28.6) | 29 (25.7) |  |
|  | A little of the time | 5 (14.3) | 18 (15.9) |  |
|  | None of the time | 11 (31.4) | 30 (26.5) |  |
| Feeling worthless; n (%) | |  |  |  |
|  | All of the time | 14 (40.0) | 38 (33.6) | 0.149 |
|  | Most of the time | 1 (2.9) | 22 (19.5) |  |
|  | Some of the time | 5 (14.3) | 19 (16.8) |  |
|  | A little of the time | 5 (14.3) | 9 (8.0) |  |
|  | None of the time | 10 (28.6) | 25 (22.1) |  |
| Kessler Total; mean (SD)* | | 19.46 (7.32) | 16.78 (6.39) | **0.038** |
| Mobility Issues; n (%) | |  |  |  |
|  | I have no problems walking | 17 (48.6) | 57 (50.4) | 0.408 |
|  | I have some problems walking | 18 (51.4) | 51 (45.1) |  |
|  | I am limited to a wheelchair or walker | 0 (0.0) | 5 (4.4) |  |
| Self-Care; n (%) | |  |  |  |
|  | I have no problems with self-care | 23 (65.7) | 93 (83.0) | 0.088 |
|  | I have some problems washing and/or dressing myself | 11 (31.4) | 17 (15.2) |  |
|  | I am unable to wash or dress myself without assistance | 1 (2.9) | 2 (1.8) |  |
| Usual Activities; n (%) | |  |  |  |
|  | I have no problems with performing my daily activities | 19 (55.9) | 76 (67.3) | 0.393 |
|  | I have some problems with performing my daily activities | 13 (38.2) | 34 (30.1) |  |
|  | I am unable to perform my activities without assistance | 2 (5.9) | 3 (2.7) |  |
| Pain/Discomfort; n (%) | |  |  |  |
|  | I have no pain or discomfort | 4 (11.4) | 35 (31.0) | 0.06 |
|  | I have moderate pain or discomfort | 16 (45.7) | 45 (39.8) |  |
|  | I have extreme pain or discomfort | 15 (42.9) | 33 (29.2) |  |
| Anxiety/Depression; n (%) | |  |  |  |
|  | I am not anxious or depressed | 13 (37.1) | 35 (31.0) | 0.167 |
|  | I am moderately anxious or depressed | 10 (28.6) | 52 (46.0) |  |
|  | I am extremely anxious or depressed | 12 (34.3) | 26 (23.0) |  |
| EQ5DL Overall; n (%) | |  |  |  |
|  | No problem | 2 (5.7) | 16 (14.2) | 0.298 |
|  | Problem | 33 (94.3) | 97 (85.8) |  |

Table 4. Differences in use and need for health and social services during the past year between individuals who consume NBA and those who do not, within a sample of individuals experiencing absolute homelessness

| Characteristics | | NBA Consumers (n=36) | Non-NBA consumers (n=113) | Significance |
| --- | --- | --- | --- | --- |
| Hospital Care; n (%) | |  |  |  |
|  | Yes, I have received care in the past 12 months | 17 (48.6) | 35 (31.2) | 0.233 |
|  | No, but I think I needed this kind of help, but did not seek it | 7 (20.0) | 22 (19.6) |  |
|  | No, I did not need this kind of help | 11 (31.4) | 54 (48.2) |  |
|  | Refused to answer | 0 (0.0) | 1 (0.9) |  |
| Counselling; n (%) | |  |  |  |
|  | Yes, I have received care in the past 12 months | 8 (22.9) | 35 (31.0) | 0.647 |
|  | No, but I think I needed this kind of help, but did not seek it | 17 (48.6) | 46 (40.7) |  |
|  | No, I did not need this kind of help | 10 (28.6) | 30 (26.5) |  |
|  | Refused to answer | 0 (0.0) | 2 (1.8) |  |
| Skills Training; n (%) | |  |  |  |
|  | Yes, I have received care in the past 12 months | 3 (8.6) | 27 (23.9) | 0.161 |
|  | No, but I think I needed this kind of help, but did not seek it | 18 (51.4) | 40 (35.4) |  |
|  | No, I did not need this kind of help | 14 (40.0) | 45 (39.8) |  |
|  | Refused to answer | 0 (0.0) | 1 (0.9) |  |
| Harm Reduction; n (%)* | |  |  |  |
|  | Yes, I have received care in the past 12 months | 11 (31.4) | 59 (52.2) | **0.003** |
|  | No, but I think I needed this kind of help, but did not seek it | 6 (17.1) | 3 (2.7) |  |
|  | No, I did not need this kind of help | 18 (51.4) | 51 (45.1) |  |
